# Interactions between corticotropin releasing factor signaling and prophylactic antibiotics on measures of intestinal function in weaned and transported pigs

**DOI:** 10.3389/fphys.2023.1266409

**Published:** 2023-10-12

**Authors:** Betty R. McConn, Kouassi R. Kpodo, Jean E. Rivier, Dominic P. Behan, Brian T. Richert, John S. Radcliffe, Donald C. Lay, Jay S. Johnson

**Affiliations:** ^1^ Oak Ridge Institute for Science and Education (ORISE), Oak Ridge, TN, United States; ^2^ Purdue University, West Lafayette, IN, United States; ^3^ Sentia Medical Sciences Inc, San Diego, CA, United States; ^4^ Livestock Behavior Research Unit, Agricultural Research Service (USDA), West Lafayette, IN, United States

**Keywords:** antibiotics, corticotropin releasing factor, intestinal physiology, pigs, swine

## Abstract

The study objective was to evaluate the interaction between corticotrophin releasing factor (**CRF**) receptor signaling and prophylactic antibiotic administration on intestinal physiology in newly weaned and transported pigs. Pigs (n = 56; 5.70 ± 1.05 kg) were weaned (20.49 ± 0.64 d), a blood sample was taken, and then pigs were given an intraperitoneal injection of saline (**SAL**; n = 28 pigs) or a CRF receptor antagonist (**CRFA**; n = 28 pigs; 30 μg/kg body weight; Astressin B), and then were transported in a livestock trailer for 12 h and 49 min. A second and third intraperitoneal injection was given at 4 h 42 min and 11 h 36 min into the transport process, respectively. Following transport, 4 SAL and 4 CRFA pigs were blood sampled and euthanized. The remaining 48 pigs were individually housed and given dietary antibiotics [**AB**; n = 12 SAL and 12 CRFA pigs; chlortetracycline (441 ppm) + tiamulin (38.6 ppm)] or no dietary antibiotics (**NAB**; n = 12 SAL and 12 CRFA pigs) for 14 d post-transport. Blood was collected at 12 h and on d 3, 7, and 14, and then pigs were euthanized on d 7 (n = 24) and d 14 (n = 24) post-weaning and transport. Circulating cortisol was reduced (*p* = 0.05) in CRFA pigs when compared to SAL pigs post-weaning and transport. On d 7, jejunal villus height and crypt depth was greater overall (*p* < 0.05) in AB-fed pigs versus NAB-fed pigs. On d 14, ileal crypt depth was reduced (*p* = 0.02) in CRFA pigs when compared to SAL pigs. Jejunal CRF mRNA abundance tended to be reduced (*p* = 0.09) on d 7 in CRFA pigs versus SAL pigs. On d 14, jejunal tumor necrosis factor-alpha was reduced (*p* = 0.01) in AB-fed pigs versus NAB-fed pigs. On d 7, change in glucose short-circuit current tended to be increased (*p* = 0.07) in CRFA pigs fed the AB diet when compared to CRFA pigs fed the NAB diet. In conclusion, CRFA pigs and pigs fed AB had some similar biological intestinal function measures post-weaning and transport.

## 1 Introduction

The weaning and transport process has the potential to induce stress and cause intestinal dysfunction in pigs due to feed withdrawal, changes in social hierarchy that lead to fighting, moving into a new environment, handling, maternal separation, and increased disease pressure (de Groot et al., 2001; Toscano et al., 2007; Remience et al., 2008; Lallès and David, 2011; Merlot et al., 2011; Ott et al., 2014). As a result, post-weaning growth performance is often reduced (Randolph et al., 1981; Hyung et al., 1998), disease incidence is increased (Wirtz et al., 2007; Song et al., 2014), and morbidity and mortality can occur (Jayaraman and Nyachoti, 2017). Several mitigation strategies have been investigated and implemented to improve post-weaning and transport growth performance and overall pig health and intestinal function. These can include management strategies (e.g., environmental management, weaning age, *etc.*), dietary manipulations (e.g., corn particle size, palatability, *etc.*), feed additives (e.g., L-glutamine, fatty acids, *etc.*), and the provision of prophylactic dietary antibiotics (Smith et al., 2010; Duttlinger et al., 2019; Figueroa et al., 2019; Modina et al., 2019; Puls et al., 2019; McConn et al., 2020; Wensley et al., 2021). However, despite these strategies, weaning and transport continue to hinder pig growth. Therefore, an improved understanding of the mechanisms underlying weaning and transport-induced intestinal dysfunction is required to further improve upon and develop more effective mitigation strategies^1^ ^,^ ^2^ ^,^ ^3^.

Intestinal dysfunction in weaned and/or transported pigs is characterized by villus atrophy (Moeser et al., 2007a; Smith et al., 2010), reduced brush-border enzyme activity (Edelblum and Turner, 2009; Groschwitz and Hogan, 2009; Turner, 2009; Marchiando et al., 2010), and greater intestinal permeability (Barbara, 2006; Camilleri and Gorman, 2007; Edelblum and Turner, 2009; Marchiando et al., 2010). Although the mechanisms underlying weaning and transport-induced intestinal dysfunction are multifactorial and interrelated (Moeser et al., 2017), previous research indicates that stress induced corticotrophin releasing factor (**CRF**) signaling in the intestine may play a role (Moeser et al., 2007a; Smith et al., 2010). This dysfunction is thought to be mediated by CRF binding to CRF receptor 1 (**CRFR1**) and CRF receptor 2 (**CRFR2**) in the intestines (Taché et al., 2004), with CRFR1 activation resulting in more negative downstream effects on intestinal function (i.e., intestinal secretion, visceral hypersensitivity, and motility changes; Maillot et al., 2000; Taché et al., 2002; Million et al., 2003; Schwetz et al., 2005). During stressful events, CRF is released from the hypothalamus which activates CRF signaling in the intestines. As a result, mast cell counts and tumor necrosis factor alpha (**TNFα**) are increased, which may mediate increased intestinal permeability (Moeser et al., 2007a; Smith et al., 2010; Pohl et al., 2017). Although it is known that CRF plays a direct role in mediating intestinal dysfunction in newly weaned pigs under controlled conditions (Moeser et al., 2007a; Smith et al., 2010), it is currently unknown whether this response occurs under production relevant conditions or whether CRF signaling interacts with commonly used dietary mitigation strategies (e.g., prophylactic antibiotics).

The study objectives were 1) to evaluate the effects of CRF receptor signaling on measures of intestinal function in weaned and transported pigs, and 2) to evaluate the interaction between CRF receptor signaling and a prophylactic antibiotic combination commonly provided following weaning and transport in the United States [chlortetracycline (Aureomycin, Zoetis, Parsippany, NJ) + tiamulin (Denagard, Elanco Animal Health, Greenfield, IN); Puls et al., 2019] on measures of intestinal function in newly weaned and transported pigs. Based upon previous research (Moeser et al., 2007a; Smith et al., 2010), we hypothesized that CRF receptor signaling would mediate weaning and transport-induced intestinal dysfunction under production relevant conditions and that blocking CRF receptor signaling during the weaning and transport process would improve measures of intestinal function similarly to the administration of commonly provided prophylactic antibiotics following weaning and transport.

## 2 Materials and methods

### 2.1 Animals and CRF receptor signaling treatments

All procedures involving animals were approved by the Purdue University Animal Care and Use Committee (protocol # 1904001875), and animal care and use standards were based upon the *Guide for the Care and Use of Agricultural Animals in Research and Teaching* (Federation of Animal Science Societies, 2010). Fifty-six crossbred pigs [(Landrace x Yorkshire) x Duroc; 5.70 ± 1.05 kg body weight (**BW**); 50% castrated males and 50% females] were weaned at 20.49 ± 0.64 d of age and then transported in central Indiana. All males were castrated within 24–48 h post-birth per normal swine production practices. One day prior to weaning and transport, all pigs were individually weighed and assigned to either an intraperitoneal saline injection treatment (**SAL**; n = 28 pigs; 30 μL/kg BW) or an intraperitoneal CRFR1/CRFR2 antagonist injection treatment (**CRFA**; n = 28 pigs; 30 μg/kg BW and injected at 30 μL/kg BW; Astressin B), balanced by BW and sex. The CRFA was obtained from J. E. F. Rivier (J. Rivier, Peptide Biology Laboratories, Salk Institute, La Jolla, CA; Sentia Medical Sciences Inc., La Jolla, CA) as previously described (Smith et al., 2010) and diluted using double sterile H_2_O. The CRFA injection dose and schedule was based upon results from a preliminary study by our group demonstrating that injecting Astressin B at a concentration of 30 μg/kg BW in 6–8 h intervals was effective in suppressing circulating adrenocorticotrophic hormone (**ACTH**; data not shown). These data confirmed previous research using Astressin B in weaned pigs (Smith et al., 2010).

### 2.2 Transportation phase

The transportation process used in the present study was previously described (Duttlinger et al., 2019; McConn et al., 2020). Briefly, on the day of weaning and transport, selected pigs were removed from sows, weighed, given an intraperitoneal injection of either SAL or CRFA, and then all pigs were loaded onto a livestock trailer (1.74 × 1.98 m; Alum-Line, Inc., Cresco, IA) using a 1.93 m long loading ramp with a 9.0° incline (maximum recommended incline is 20.0°; National Pork Board, 2015). The trailer provided 0.062 m^2^ space per pig, which was within the range required for 4.54–9.07 kg BW pigs (Federation of Animal Science Societies, 2010). Within the trailer, two data loggers (Hobo data logger temperature/RH; accuracy ±0.20°C; Onset; Bourne, MA) were evenly spaced approximately 1.22 m above pig level to measure ambient temperature (**T**_**A**_) and relative humidity (**RH**) in 5-min intervals. During transport, T_A_ and RH were 26.93°C ± 0.02°C and 62.25% ± 0.09%, respectively. Blood samples (5 mL) were collected via jugular venipuncture immediately prior to transport and immediately post-transport for all pigs using 5 mL K2 EDTA tubes (BD vacutainers; Franklin Lakes, NJ).

Group transport covered a distance of 836 km, and feed and water were withheld per normal production practices (Hicks et al., 1998). Total group transport time was 12 h 49 min and this included loading time, time spent in the trailer, injection time, unloading time, and the time it took to be sorted into their respective pens. Specifically, the average time to wean pigs, take blood, administer the first injection treatment, and load the pigs into the trailer was 1 h 25 min. The transport route was 279 km in length and took approximately 3 h 17 min. This route was completed 3 times and following the completion of each route the driver was switched, and the truck was refueled. The second injection was administered after the first route completion (4 h 42 min into the transport process) and this process took 30 min. At the conclusion of transport, pigs were unloaded from the trailer, blood was taken, the third injection treatment was administered (11 h 36 min into the transport process), and then pigs were individually weighed and placed into their respective individual pens. This process took a total of 1 h 3 min.

### 2.3 Sentinel pigs

Eight sentinel pigs (n = 2 pigs/injection treatment/sex) were used to obtain descriptive data following the weaning and transport procedure. Immediately following the transport procedure (**12 h Post-T**), 4 sentinel pigs (n = 1 pig/injection treatment/sex) were euthanized by CO_2_ exposure and exsanguination for jejunal and ileal tissue collection. The remaining 4 sentinel pigs (n = 1 pig/injection treatment/sex) were housed at thermoneutral conditions (26.11°C ± 0.04°C; 39.12% ± 0.10% RH) in a group pen (1.22 m × 1.37 m) and provided antibiotic-free feed and water *ad libitum*. At 24 h post-weaning (**24 h Post-T**), blood samples (5 mL) were collected via jugular venipuncture using 5 mL K2 EDTA tubes (BD vacutainers; Franklin Lakes, NJ) and then the pigs were euthanized at 24 h post-weaning by CO_2_ exposure and exsanguination for jejunal and ileal tissue collection. Two portions of the jejunum (starting approximately 0.7 m distal to the pyloric sphincter) and 2 portions of the ileum (starting approximately 0.2 m anterior to the ileal-cecal junction) were collected from each pig. One jejunal and ileal section was placed into 10% formalin for histology and one jejunal and ileal section was snap-frozen in liquid nitrogen and stored at −80°C for later mRNA extraction.

### 2.4 Dietary treatment phase

Following transport, 24 SAL and 24 CRFA pigs, were randomly allotted to individual pens (0.83 m × 0.71 m) and housed under thermoneutral conditions (28.97°C ± 0.06°C; 41.27% ± 0.12% RH) based on temperature guidelines for newly weaned pigs (Federation of Animal Science Societies, 2010) for 14 d. Pigs were allotted to 1 of 2 dietary treatments, balanced by injection treatment, pre-transport BW, and sex, and fed for 14 d in 2 phases (d 0 to 7 and 7 to 14 post-weaning and transport; Supplementary Table S1). Diets were corn-soybean meal-based, fed in meal form, and were formulated to meet or exceed nutrient requirements (NRC, 2012) during the nursery period (Supplementary Table S1). Dietary treatments were antibiotics [**AB**; n = 12 SAL and 12 CRFA pigs; chlortetracycline (441 ppm) + tiamulin (38.6 ppm)] or no antibiotics (**NAB**; n = 12 SAL and 12 CRFA pigs). Treatment diets and water were provided *ad libitum* for 14-d post-transport based on typical production practices for AB administration (Veterinary Feed Directive, 2017). Body weight and feed intake were measured on d 0, 7, and 14 at 0700 h to calculate average daily BW gain (**ADG**), average daily feed intake (**ADFI**), and feed efficiency (**ADG:ADFI**). Blood samples (5 mL) were collected via jugular venipuncture using 5 mL K2 EDTA tubes (BD vacutainers; Franklin Lakes, NJ) at 0700 h on d 3, 7, and 14. On d 7 and 14 post-weaning and transport, 24 pigs (evenly distributed by injection treatment, dietary treatment, and sex) were euthanized by CO_2_ exposure and exsanguination, respectively. Tissues were collected and processed as previously described in Section 2.3. Additionally, a third portion of the jejunum and ileum was collected and placed in a modified Krebs buffer solution (25 mM NaHCO_3_, 118 mM NaCl, 4.7 mM KCl, 1.2 mM MgSO_4_, 1.2 mM NaH_2_PO_4_, and 1.2 mM CaCl_2_; pH 7.4). The samples placed in the modified Krebs buffer solution were used for *ex vivo* intestinal permeability measurements with modified Ussing chambers (Physiologic Instruments, Inc., San Diego, CA) as previously described (Saddoris et al., 2019; Kpodo et al., 2020).

### 2.5 Animal behavior

As previously described in detail by Duttlinger et al. (2019), pigs were video recorded 24 h/d throughout the entire 14-d experiment using ceiling-mounted cameras (Panasonic WV-CP254H, Matsushita Electric Industrial Co. Ltd., Osaka, Japan) immediately following weaning and transport. Video files were recorded using a digital video recorder system (GeoVision VMS Software; GeoVision Inc., Tapei, Taiwan). Two trained individuals who were blind to the treatments recorded the individual behaviors using an instantaneous scan sampling technique in 10-min intervals (Duttlinger et al., 2019) on d 2, 4, 8, and 12 post-weaning and transport for three periods each day (0600–1,000 h, 1,100–1,500 h, and 1,600–2000 h). Agreement between the two trained individuals was 92%. Behavioral analysis days were selected so that they did not fall on days in which pigs were being handled or blood samples were being taken. Specific recorded behaviors included posture (lying, standing, sitting) and consumption (eating, drinking, other) and are described in an ethogram (Table 1).

**TABLE 1:** Ethogram used for behavioral analyses.

Behavior	Definition
Posture	
Lying	Body in contact with floor, either sternally or laterally
Standing	Standing on all four legs
Sitting	Positioned with rump on floor and hindlegs underneath
Nonvisible	Moved out of view and cannot be observed
Consumption	
Eating	Head positioned in the feeder
Drinking	Snout in contact with the waterer
Other	Anything other than eating or drinking
Nonvisible	Moved out of view and cannot be observed

### 2.6 Blood parameters

Plasma tube samples were centrifuged at 4°C and 1,900 x *g* for 15 min, aliquoted and stored at −80°C for further analysis. Circulating cortisol concentrations were analyzed using a commercially available radioimmunoassay (**RIA**) kit (minimum detectable level: 0.9 μg/mL; Cortisol RIA, IBL International, Hamburg, Switzerland) according to the manufacturer’s instructions. Plasma non-esterified fatty acid (**NEFA**) concentrations were analyzed using a commercially available colorimetric assay kit (minimum detectable level: 125 µEq/L; HR Series NEFA-HR 2), Fujifilm Wako Diagnostics, Mountain View, CA) according to the manufacturer’s instructions. Using a commercially available enzyme-linked immunosorbent assay (**ELISA**) kit (minimum detectable level: 1.37 ng/mL; Bethyl Laboratories, Inc., Montgomery, TX), plasma immunoglobulin A (**IgA**) concentrations were analyzed. A commercially available colorimetric assay kit (minimum detectable level: 25 mg/dL; Autokit Glucose, Fujifilm Wako Diagnostics, Mountain View, CA) was used to determine plasma glucose concentrations. Plasma insulin concentrations were analyzed using a commercially available ELISA kit (minimum detectable level: 1.15 mU/L; Porcine Insulin ELISA, Mercodia, Uppsala, Sweden). Using a commercially available ELISA kit (minimum detectable level: 9.38 pg/mL; Pig ACTH ELISA kit, Abbexa, Houston, TX), plasma adrenocorticotropic hormone (**ACTH**) concentrations were analyzed. Prior to performing the lipopolysaccharide (**LPS**) analysis, plasma was diluted 1:2 with Endotoxin-Free water and placed in a water bath for 15 min. Samples were then centrifuged at 4°C and 1900 x *g* for 15 min and analyzed using a commercially available Pierce Chromogenic Endotoxin Quant Kit (minimum detectable level: 0.01 EU/mL; Thermo Fisher Scientific, Waltham, MA). Plasma samples were also submitted to the University of Minnesota Cytokine Reference Laboratory for cytokines (interleukin (**IL**)-1 alpha, IL-1α; IL-1β; IL-6; IL-8; tumor necrosis factor alpha, **TNFα**) analyses using a multiplex assay. The intra-assay and inter-assay coefficients of variation for all the blood parameters were less than 10% and 20%, respectively, as suggested by the manufacturer.

### 2.7 Intestinal morphology

Histological analyses were performed as previously described (Johnson et al., 2016; Kpodo et al., 2020). Briefly, jejunal and ileal samples were placed in 10% formalin solution and referred to the Purdue University Histology and Phenotyping Laboratory to be sectioned (5-µm thickness) and stained (Acian blue and Giemsa). A random location on the slide was selected to be magnified at 4 or 10 X depending on villi height and imaged using Q-capture Pro 6.0 software (Qimaging, Surrey, British Columbia, Canada). The imaged selections were then analyzed for villus height (**VH**; µm), crypt depth (**CD**; µm), and goblet cell counts using the ImageJ 1.47v software (National Institutes of Health; Bethesda, MD). The VH:CD ratio was calculated by dividing the VH by CD. Goblet cell count was divided by the corresponding VH to calculate the goblet cell count per mm of villi (Horn et al., 2009). Additionally, using a magnification of ×40, mast cell counts were obtained based on the procedure described in Kpodo et al. (2020). Prior to data analyses, histological measures were averaged on a per pig basis.

### 2.8 Ussing chambers

Jejunal integrity and nutrient transport were measured using modified Ussing chambers as previously described (Saddoris et al., 2019; Kpodo et al., 2020). Briefly, the outer serosal layer was removed and the remaining tissue (epithelial and submucosal layer) was mounted in modified Ussing chambers equipped to measure transepithelial resistance **(TER)** as well as glucose baseline Isc (**bIsc**), maximum Isc (**mIsc**), and the change in bIsc to mIsc (**dIsc**). Tissues (1.0 cm^2^ surface area) were mounted in duplicates and the chambers were filled with 8 mL modified Krebs buffer solution. Tissues were kept at 37.0°C using a circulating water bath (VWR, Batavia, IL) and were continuously aerated using carbogen gas (95% O_2_ and 5% CO_2_). After tissues were allowed to equilibrate for 30 min, glucose (10 mmol/L) was added to the mucosal side and mannitol (10 mmol/L) to the serosal side (10 mmol/L) for osmotic balance. The active transport of glucose was determined by measuring the dIsc.

### 2.9 mRNA relative abundance

Jejunal and ileal tissue were ground separately in 2 mL microtubes with a Dremel, Buffer RLT (lysis buffer; 990 µL) and β-mercaptoethanol (10 µL), and then the microtubes were centrifuged at 4°C for 3 min at maximum speed (14,000 G). After centrifugation, the supernatant was removed (700 µL), 70% ethanol was added (700 µL), and the mixture was transferred to a spin column and total RNA was purified using the RNeasy Mini Kit (QIAGEN, Germantown, MD). First-strand cDNA was synthesized from 100 ng total RNA in 20 μL reactions with a High Capacity cDNA Reverse Transcription Kit (Applied Biosystems, Foster City, CA) following the manufacturer’s instructions. Reactions were performed under the following conditions: 25°C for 10 min, 37°C for 120 min and 85°C for 5 min. Real-time PCR reactions were performed in 10 µL reactions and contained 5 µL Fast SYBR Green Master Mix (Applied Biosystems, Foster City, CA), 0.5 µL primers (forward and reverse), 1.5 µL nuclease-free water, and 3 µL 10-fold diluted cDNA using a Step One Plus Real-time PCR System (Applied Biosystems, Foster City, CA). The real-time PCR was performed under the following conditions: 95°C for 20 s, 40 cycles of 95°C for 3 s, and 60°C for 30 s. Primers for real-time PCR are listed in Supplementary Table S2. Relative quantification was performed as the data were analyzed using the ΔΔCT method and GAPDH served as the reference gene. The SAL-injected NAB-fed pigs served as the calibrator sample, calculated as 2^−ΔΔCT^ were used for statistical analyses.

### 2.10 Statistics

Data were analyzed using the PROC MIXED procedure in SAS 9.4 (SAS Institute INC., Cary, NC). Individual pig was the experimental unit for all analyses. The assumptions of normality of error, homogeneity of variance, and linearity were confirmed *post hoc*. An outlier box plot was used to identify outliers for each parameter. Outliers were removed if they were outside the quantile range and not supported biologically. Sex was included in all analyses but was removed when no differences were detected. For all repeated analyses, the covariance structure was selected based on the Akaike’s Information Criteria (Littell et al., 1998) with day or week as the repeated effect as needed. All data are presented as least squares means (**LSmeans**) ± standard error (**SE**). Litter, BW block, plate, and pig were included as random effects when applicable. Statistical significance was defined as *p* ≤ 0.05 and a tendency was defined as 0.05 < *p* ≤ 0.10.

#### 2.10.1 Transport phase analyses

For analyses of blood parameter data during the transport phase, injection treatment (CRFA and SAL), collection time [pre-transport (**Pre-T**) and post-transport (**Post-T**)], and their interactions were used as fixed effects. The time it took in seconds to take blood (measured from the moment the pig was handled to the end of blood collection) was used as a covariate when significant (*p* ≤ 0.05) for plasma cortisol and ACTH levels because animal handling time directly influences ACTH and cortisol release (Hasiec et al., 2017; Rocha et al., 2019).

#### 2.10.2 Diet treatment phase analyses

Behavioral data were expressed as percentage ±SE. Injection (CRFA and SAL), diet treatment (AB and NAB), d (2, 4, 8, and 12), and their interactions were included as fixed effects.

Injection (CRFA and SAL), diet treatment (AB and NAB), week (1 and 2), and their interactions were included as fixed effects for ADG, ADFI, and ADG: ADFI analyses.

Fixed effects for blood parameter data included injection (CRFA and SAL), diet treatment (AB and NAB), d (3, 7, and 14), and their interactions. The time it took to take blood (in s) was used as a covariate when significant (*p* ≤ 0.05) for plasma cortisol and ACTH levels. IL-1α, IL-1β, IL-6, IL-8, and TNF-α were log-transformed to meet assumptions of normality; however, all log-transformed data are presented as back-transformed LSmeans ± SE for ease of interpretation.

Injection (CRFA and SAL), diet treatment (AB and NAB), and their interactions were included as fixed effects for all intestinal health measures. Jejunal CRFR1 and CRFR2 mRNA abundance in the jejunum on d 7 and 14, CRF mRNA in the jejunum and ileum on d 14, and CRFR2 mRNA in the ileum on d 14 were log-transformed to meet assumptions of normality. All remaining mRNA abundance data were square root-transformed to meet assumptions of normality. However, all transformed data are presented as back-transformed LSmeans ± SE for ease of interpretation.

## 3 Results

### 3.1 Transport phase blood characteristics

#### 3.1.1 Stress hormones

**FIGURE 1:**
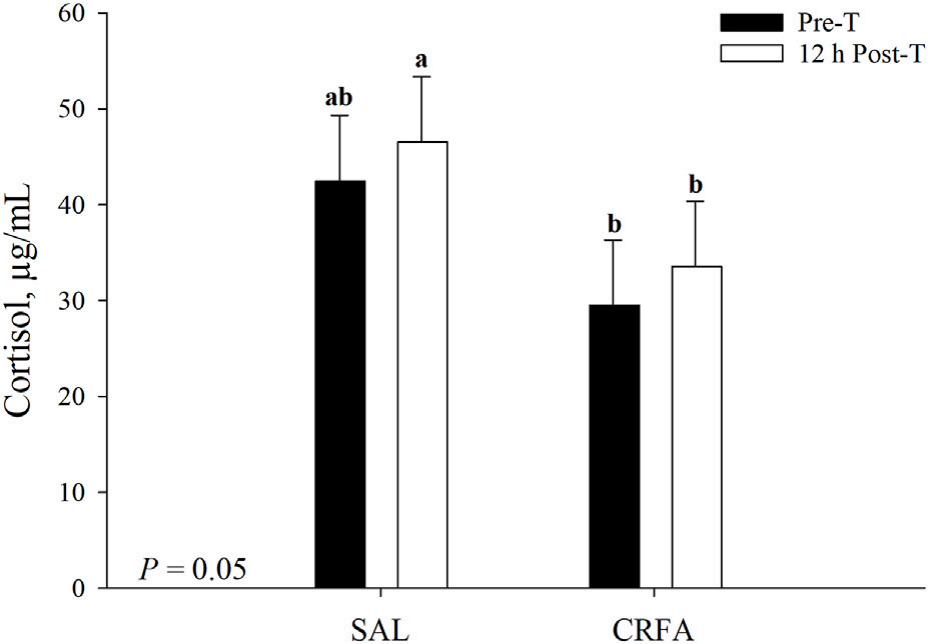
Interaction of giving pigs an intraperitoneal corticotropin releasing hormone antagonist (**CRFA**) or sterile saline (**SAL**) injection pre-weaning and transport (**Pre-T**) and 12 h post-weaning and transport (**12 h Post-T**) on plasma cortisol in pigs. Data are presented as LSmeans ±1 SE. Letters^a,b^ indicate injection treatment by collection time differences (*p* ≤ 0.05).

Circulating cortisol was reduced (*p* = 0.05) in CRFA pigs Pre-T (36.6%) and 12 h Post-T (27.9%) when compared to SAL pigs 12 h Post-T, but no differences were detected when compared to SAL pigs Pre-T (Figure 1). Cortisol was reduced (*p* < 0.01; 29.2%) in CRFA versus SAL pigs, regardless of collection time (Table 2). Moreover, cortisol was greater (*p* = 0.02; 11.3%) 12 h Post-T versus Pre-T, regardless of injection treatment (Table 2). No other stress hormone differences were detected (*p* > 0.10) during the transport phase with any comparison (Table 2).

**TABLE 2:** Effects of giving pigs an intraperitoneal corticotropin releasing hormone antagonist (CRFA) or sterile saline (SAL) injection during the weaning and transport process and then feeding a diet with no prophylactic antibiotics (NAB) or with prophylactic antibiotics (AB) for 14 d on circulating stress hormones (cortisol and ACTH), post-absorptive metabolism (NEFA, glucose, and insulin), and immune system biomarkers (IgA, LPS, IL-1α, IL-1β, IL-6, IL-8, and TNFα).

	Injection treatment		Collection time			*p*-value		
Parameter	SAL	CRFA	Pre-T^a^	12 h Post-T^b^	SEM	I^c^	T^d^	I x T
Cortisol, µg/mL	44.53	31.54	36.01	40.07	6.81	<0.01	0.02	0.05
ACTH, pg/mL	129.09	129.10	132.50	125.69	15.89	0.95	0.12	0.64
NEFA, uEq/L	480.46	480.46	400.93	559.99	31.38	0.23	<0.01	0.48
Glucose, mg/dL	114.80	114.81	120.79	108.82	3.97	0.66	<0.01	0.66
Insulin, mU/L	6.51	6.01	8.22	4.30	1.15	0.10	<0.01	0.73
IgA, mg/L	301.88	301.89	299.97	303.81	35.18	0.59	0.09	0.54
LPS, EU/mL	0.06	0.06	0.07	0.05	<0.01	0.85	<0.01	0.80
IL-1α, pg/mL	1.44	1.31	1.62	1.12	0.94	0.08	0.51	0.74
IL-1β, pg/mL	0.36	0.37	0.42	0.31	0.30	0.85	0.08	0.31
IL-6, pg/mL	0.45	0.46	0.45	0.46	0.34	0.94	0.89	0.83
IL-8, pg/mL	1.87	1.93	1.56	2.24	1.13	0.66	0.08	0.50
TNFα, pg/mL	2.24	2.10	1.87	2.47	1.64	0.15	0.55	0.48

^a^ Pre-T, immediately prior to transport.

^b^ 12 h Post-T, 12 h post-weaning and transport.

^c^ Injection treatment.

^d^ Collection Time.

#### 3.1.2 Post-absorptive metabolism hormones and metabolites

Insulin levels tended to be reduced (*p* = 0.10; 7.7%) in CRFA versus SAL pigs, regardless of collection time (Table 2). Glucose and insulin levels were reduced (*p* < 0.01; 9.9% and 47.7%, respectively) 12 h Post-T versus Pre-T, regardless of injection treatment (Table 2). Overall, NEFA levels were greater (*p* < 0.01; 39.7%) 12 h Post-T when compared to Pre-T, regardless of injection treatment (Table 2). Circulating glucose levels were greater (*p* = 0.02) in castrated males (118.54 ± 4.04 dL/mL) when compared to females (111.07 ± 3.91 dL/mL), regardless of injection treatment or collection time (data not presented). No other post-absorptive hormone and metabolite differences were detected (*p* > 0.10) during the transport phase with any comparison (Table 2).

#### 3.1.3 Immune system biomarkers

Circulating LPS was reduced (*p* < 0.01; 28.6%) 12 h Post-T versus Pre-T, regardless of injection treatment (Table 2). Immunoglobulin A tended to be greater (*p* = 0.09; 1.3%) 12 h Post-T versus Pre-T, regardless of injection treatment (Table 2). Interleukin-1α levels tended to be reduced (*p* = 0.08; 9.1%) in CRFA pigs when compared to SAL pigs, regardless of collection time (Table 2). Overall, IL-1β levels tended to be reduced (*p* = 0.08; 27.4%) and circulating IL-8 tended to be greater (*p* = 0.08; 44.4%) 12 h Post-T versus Pre-T, regardless of injection treatment (Table 2). No other immune system biomarker differences were detected (*p* > 0.10) during the transport phase with any comparison (Table 2).

### 3.2 Sentinel data

All sentinel data are for descriptive purposes only. Intestinal morphology and mRNA abundance data during 12 h Post-T and 24 h Post-T are presented in Supplementary Table S3.

### 3.3 Dietary treatment phase

#### 3.3.1 Growth performance

Average daily gain tended to be reduced overall (*p* = 0.08; 27.8%) in CRFA pigs when compared to SAL pigs from d 0 to 7, regardless of dietary treatment (Table 3). No other growth performance differences were detected (*p* > 0.10) during the dietary treatment phase with any comparison (Table 3).

**TABLE 3:** Effects of giving pigs an intraperitoneal corticotropin releasing hormone antagonist (CRFA) or sterile saline (SAL) injection during the weaning and transport process and then feeding a diet with no prophylactic antibiotics (NAB) or with prophylactic antibiotics (AB) for 14 d on postnatal growth performance parameters.

	SAL		CRFA			*p*-value		
Parameter	NAB	AB	NAB	AB	SEM	I^a^	D^b^	I x D
Days 0–7								
d 0 BW, kg	5.94	6.00	5.96	5.99	0.48	0.87	0.28	0.79
ADG, g/d	107	87	79	61	26	0.08	0.63	0.90
ADFI, g/d	180	145	153	172	25	0.19	0.48	0.62
ADG:ADFI, kg/kg	0.59	0.35	0.47	0.50	0.36	0.12	0.77	0.85
d 7 BW, kg	6.71	6.33	6.44	6.60	0.63	0.18	0.46	0.86
Days 8–14								
ADG, g/d	267	226	237	256	57	0.47	0.74	0.23
ADFI, g/d	459	438	463	434	79	0.80	0.73	0.99
ADG:ADFI, kg/kg	0.58	0.52	0.52	0.57	0.06	0.50	0.59	0.15
d 14 BW, kg	8.65	8.63	8.49	8.80	1.62	0.97	0.61	0.46

^a^ Injection treatment.

^b^ Diet treatment.

#### 3.3.2 Behavior

No behavioral differences (*p* > 0.10) were detected during the dietary treatment phase with any comparison (Table 4).

**TABLE 4:** Effects of giving pigs an intraperitoneal corticotropin releasing hormone antagonist (CRFA) or sterile saline (SAL) injection during the weaning and transport process and then feeding a diet with no prophylactic antibiotics (NAB) or with prophylactic antibiotics (AB) for 14 d on behavior in pigs.

	SAL		CRFA			*p*-value		
Parameter	NAB	AB	NAB	AB	SEM	I^a^	D^b^	I x D
Posture								
Lying, %	75.30	77.13	73.00	76.90	5.31	0.78	0.17	0.84
Standing, %	9.14	10.58	10.71	10.90	0.83	0.12	0.87	0.33
Sitting, %	8.58	7.70	5.52	8.14	0.55	0.87	0.29	0.70
Nonvisible, %	6.98	4.59	10.77	4.06	1.65	0.73	0.11	0.35
Consumption								
Eating, %	8.70	9.36	9.02	9.85	0.87	0.21	0.21	0.67
Drinking, %	5.00	4.96	5.37	5.12	0.35	0.86	0.76	0.80
Other, %	83.09	83.25	81.64	81.42	3.29	0.72	0.88	0.72
Nonvisible, %	3.21	2.43	3.97	3.61	0.57	0.34	0.41	0.46

^a^ Injection treatment.

^b^ Diet treatment.

#### 3.3.3 Stress hormones

**FIGURE 2:**
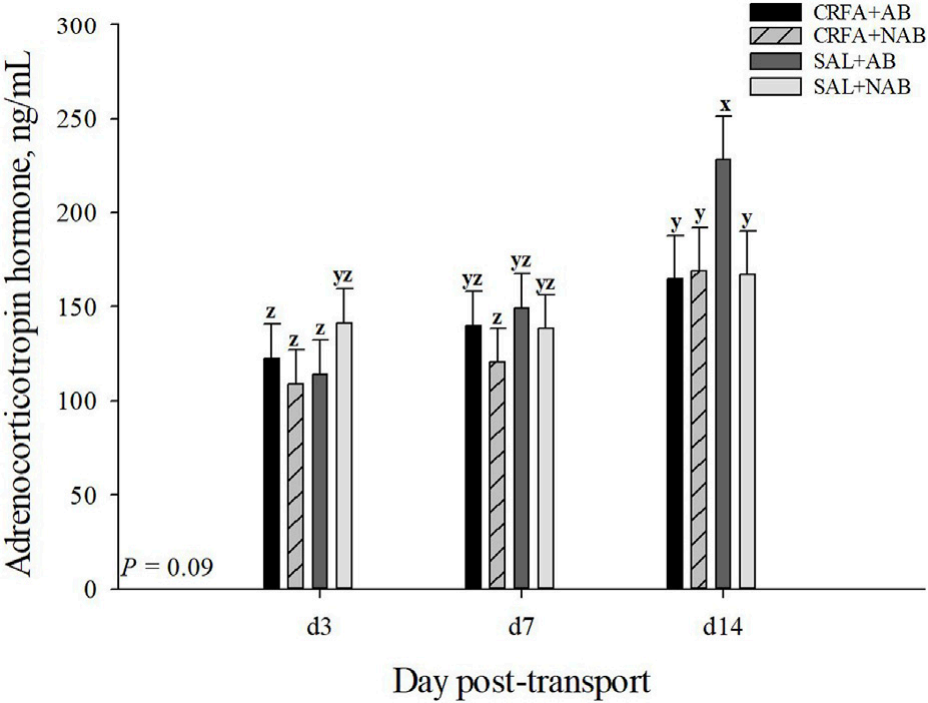
Effects of giving pigs an intraperitoneal corticotropin releasing hormone antagonist (**CRFA**) or sterile saline (**SAL**) injection on plasma adrenocorticotropic hormone in pigs fed no prophylactic antibiotics (**NAB**) or prophylactic antibiotics (**AB**) following weaning and transport on d 3, 7, and 14 during the dietary treatment phase. Data are presented as LSmeans ±1 SE. Letters^x-z^ indicate injection treatment by diet treatment by day tendencies (0.05 < *p* ≤ 0.10).

An injection treatment by diet treatment by collection time effect was observed where on d 14, ACTH levels tended to be greater (*p* = 0.09) in SAL pigs fed the AB diet when compared to all other treatment groups on d 3, 7, and 14 (Figure 2). On d 14, CRFA pigs fed the AB diet, CRFA pigs fed the NAB diet, and SAL pigs fed the NAB diet tended to have greater ACTH levels (*p* = 0.09) versus CRFA pigs fed the AB diet, CRFA pigs fed the NAB diet, and SAL pigs fed the AB diet on d 3 and CRFA pigs fed the NAB diet on d 7 (Figure 2). Overall, ACTH levels tended to be reduced (*p* = 0.06; 12.0%) in CRFA pigs versus SAL pigs, regardless of dietary treatment or collection time (Table 5). No other stress hormone differences were detected (*p* > 0.10) during the dietary treatment phase with any comparison (Table 5; Figure 2).

**TABLE 5:** Effects of injection treatment on blood parameters of stress hormones (cortisol and ACTH), post-absorptive metabolism (NEFA, glucose, and insulin), and immune system biomarkers (IgA, LPS, IL-1α, IL-1β, IL-6, IL-8, and TNFα) in pigs given an intraperitoneal corticotropin releasing hormone antagonist (CRFA) or sterile saline (SAL) injection during the weaning and transport process and then fed a diet with no prophylactic antibiotics (NAB) or with prophylactic antibiotics (AB) for 14 d.

	SAL		CRFA			*p*-value		
Parameter	NAB	AB	NAB	AB	SEM	I^a^	D^b^	I x D
Cortisol, µg/mL	46.18	41.42	46.72	40.88	7.96	0.58	0.25	0.32
ACTH, pg/mL	148.97	163.94	132.79	142.45	13.35	0.06	0.21	0.79
NEFA, uEq/L	317.06	394.55	404.39	307.21	101.04	0.41	0.28	0.44
Glucose, mg/dL	106.19	103.20	105.96	103.44	4.88	0.49	0.55	0.34
Insulin, mU/L	6.00	4.70	6.16	4.54	0.79	0.25	0.15	0.69
IgA, mg/L	495.28	429.30	550.57	460.39	77.74	0.32	0.07	0.78
LPS, EU/mL	0.05	0.06	0.05	0.06	0.01	0.28	0.86	0.26
IL-1α, pg/mL	1.29^y^	1.14^xy^	1.21^xy^	1.07^x^	0.78	0.70	0.72	0.07
IL-1β, pg/mL	0.24	0.37	0.29	0.31	0.21	0.34	0.90	0.58
IL-6, pg/mL	0.17	0.28	0.19	0.35	0.13	0.04	0.03	0.13
IL-8, pg/mL	2.19^b^	1.86^ab^	1.82^a^	2.29^b^	0.41	0.68	0.58	0.05
TNFα, pg/mL	0.78	0.87	1.74	1.82	0.99	0.05	0.11	0.48

^a^ Injection treatment.

^b^ Diet treatment.

^a,b^Letters indicate significance (*p* ≤ 0.05) within a row and treatment interaction.

^x,y^Letters indicate tendencies (0.05 < *p* ≤ 0.10) within a row and treatment interaction.

#### 3.3.4 Post-absorptive hormones and metabolites

No post-absorptive hormone and metabolite differences were detected (*p* > 0.10) during the dietary treatment phase with any comparison (Table 5).

#### 3.3.5 Immune system biomarkers

**FIGURE 3:**
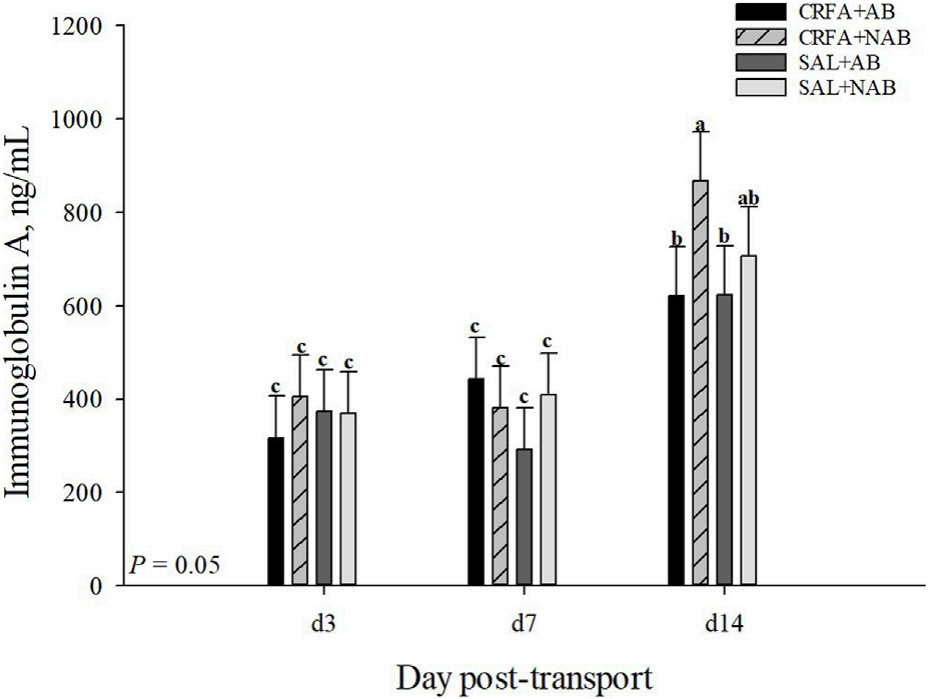
Effects of giving pigs an intraperitoneal corticotropin releasing hormone antagonist (**CRFA**) or sterile saline (**SAL**) injection on plasma Immunoglobin A in pigs fed no prophylactic antibiotics (**NAB**) or prophylactic antibiotics (**AB**) following weaning and transport on d 3, 7, and 14 during the dietary treatment phase. Data are presented as LSmeans ±1 SE. Letters^a-c^ indicate injection treatment by diet treatment by day differences (*p* ≤ 0.05).

**FIGURE 4:**
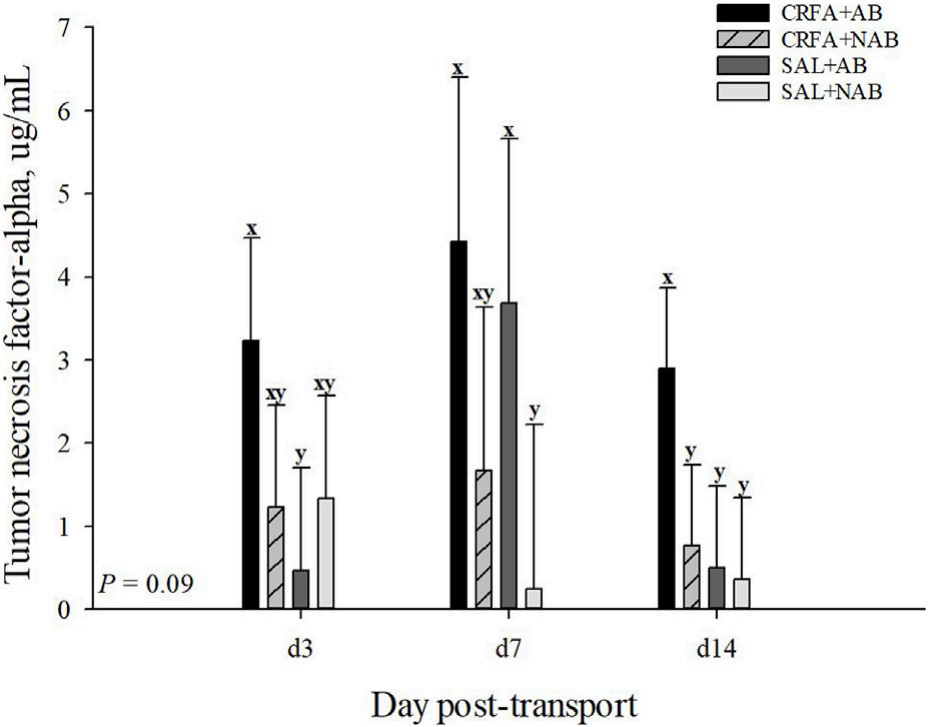
Effects of giving pigs an intraperitoneal corticotropin releasing hormone antagonist (**CRFA**) or sterile saline (**SAL**) injection on plasma tumor necrosis factor-alpha in pigs fed no prophylactic antibiotics (**NAB**) or prophylactic antibiotics (**AB**) following weaning and transport on d 3, 7, and 14 during the dietary treatment phase. Data are presented as LSmeans ±1 SE. Letters^x-y^ indicate injection treatment by diet treatment by day tendencies (0.05 < *p* ≤ 0.10).

An injection treatment by diet treatment by collection time effect was observed for IgA where on d 14, CRFA pigs fed the NAB diet had greater (*p* = 0.05) IgA levels when compared to all other treatments on d 3, 7, and 14, except for SAL pigs fed NAB diet on d 14 (Figure 3). On d 14, CRFA pigs fed the AB diet, SAL pigs fed the AB diet, and SAL pigs fed the NAB diet had greater (*p* = 0.05) IgA levels versus all other treatments on d 3 and 7 (Figure 3). Moreover, an injection treatment by diet treatment by collection time effect was observed for TNFα where CRFA pigs fed the AB diet on d 3, 7, and 14 tended to have greater (*p* = 0.09) circulating TNFα versus SAL pigs fed the NAB diet on d 7 and 14 (Figure 4). On d 7, SAL pigs fed the AB diet tended to have greater (*p* = 0.09) circulating TNFα when compared to SAL pigs fed the AB diet on d 3 and 14 (Figure 4). Additionally, on d 7, SAL pigs fed the AB diet tended to have greater (*p* = 0.09) circulating TNFα versus CRFA pigs fed the NAB diet and SAL pigs fed the NAB diet on d 14 (Figure 4). Overall, IL-1α tended to be reduced (*p* = 0.07; 17.1%) in CRFA pigs fed the AB diet when compared to SAL pigs fed the NAB diet, regardless of collection time (Table 5). In addition, IL-8 was greater (*p* = 0.05; 23.1%) in SAL pigs fed NAB diet and CRFA pigs fed AB diet versus CRFA pigs fed NAB diet, regardless of collection time (Table 5). Overall, IgA tended to be reduced (*p* = 0.07; 14.9%) in AB-fed pigs when compared to NAB-fed pigs, regardless of injection treatment or collection time (Table 5). Additionally, IL-6 was greater (*p* = 0.03; 75.0%) in AB-fed pigs versus NAB-fed pigs, regardless of injection treatment or collection time (Table 5). Moreover, IL-6 and TNFα were greater (*p* = 0.04 and 0.05, respectively; 20.0% and 115.8%, respectively) in CRFA pigs when compared to SAL pigs, regardless of dietary treatment or collection time (Table 5). No other immune system biomarker differences were detected (*p* > 0.10) during the dietary treatment phase with any comparison (Table 5; Figures 3, 3).

#### 3.3.6 Intestinal morphology

On d 7, jejunal VH and CD was greater (*p* = 0.04 and 0.02, respectively; 22.5% and 14.4%, respectively) in AB-fed pigs when compared to NAB-fed pigs, regardless of injection treatment (Table 6). Ileal VH:CD tended to be greater (*p* = 0.08; 6.7%) in AB-fed pigs versus NAB-fed pigs on d 7, regardless of injection treatment (Table 6). On d 14, ileal CD was reduced (*p* = 0.02; 5.1%) in CRFA pigs when compared to SAL pigs, regardless of dietary treatment (Table 6). A sex difference was observed where jejunal goblet cells tended to be reduced (*p* = 0.06) in castrated males (3.36 ± 0.80) when compared to females (5.30 ± 0.76) on d 7, regardless of injection and diet treatment (data not presented). No other intestinal morphology parameter differences were detected (*p* > 0.10) during the dietary treatment phase with any comparison (Table 6).

**TABLE 6:** Effects of giving pigs an intraperitoneal corticotropin releasing hormone antagonist (CRFA) or sterile saline (SAL) injection during the weaning and transport process and then feeding a diet with no prophylactic antibiotics (NAB) or with prophylactic antibiotics (AB) on intestinal morphology parameters on d 7 and 14 post-weaning and transport.

	SAL		CRFA			*p*-value		
Parameter	NAB	AB	NAB	AB	SEM	I^a^	D^b^	I x D
*Day 7*								
Jejunum								
Villus height, µm	405.31	457.32	370.27	492.36	38.46	0.35	0.04	0.87
Crypt depth, µm	190.31	203.63	177.17	216.77	10.85	0.40	0.02	0.95
VH:CD^c^	2.23	2.14	2.07	2.30	0.12	0.54	0.11	0.98
Goblet cells, cell/mm^2^	4.50	4.15	4.43	4.22	0.74	0.67	0.80	0.50
Mast cells, cell/mm^2^	8.18	8.04	9.15	7.07	1.37	0.92	0.12	0.45
Ileum								
Villus height, µm	342.94	360.12	328.84	374.22	22.13	0.59	0.14	0.78
Crypt depth, µm	168.69	180.67	174.84	174.52	5.39	0.13	0.97	0.50
VH:CD	1.99	2.00	1.87	2.12	0.14	0.92	0.08	0.97
Goblet cells, cell/mm^2^	13.12	12.32	11.18	14.26	1.53	0.72	0.17	0.50
Mast cells, cell/mm^2^	11.20	11.15	10.59	11.76	1.37	0.97	0.45	0.53
*Day 14*								
Jejunum								
Villus height, µm	523.67	492.40	511.10	504.97	52.94	0.61	0.91	0.40
Crypt depth, µm	234.74	229.26	231.22	232.74	13.39	0.69	0.91	0.74
VH:CD	2.21	2.11	2.18	2.15	0.09	0.45	0.80	0.15
Goblet cells, cell/mm^2^	6.26	6.19	6.35	6.09	0.99	0.96	0.83	0.75
Mast cells, cell/mm^2^	6.39	7.86	7.47	6.79	0.71	0.17	0.49	0.14
Ileum								
Villus height, µm	392.53	372.20	380.94	383.79	31.34	0.65	0.94	0.33
Crypt depth, µm	216.35	206.20	205.55	195.40	15.11	0.02	0.91	0.80
VH:CD	1.94	1.95	1.93	1.97	0.10	0.96	0.73	0.63
Goblet cells, cell/mm^2^	10.14	9.56	9.15	10.55	2.05	0.70	0.29	0.58
Mast cells, cell/mm^2^	15.23	13.66	15.46	13.43	1.41	0.31	0.12	0.66

^a^ Injection treatment.

^b^ Diet treatment.

^c^ Villus height: Crypt depth.

#### 3.3.7 Intestinal mRNA relative abundance

On d 7, jejunal ZO-1 mRNA abundance tended to be greater (*p* = 0.09; 60.0%) in CRFA pigs fed the AB diet when compared to SAL pigs fed the NAB diet (Table 7). Jejunal CLAD mRNA abundance tended to be reduced (*p* = 0.06; 49.5%) in CRFA pigs fed the AB diet when compared to SAL pigs fed the NAB diet and CRFA pigs fed the NAB diet on d 7 (Table 7). In addition, jejunal CRFR2 mRNA abundance tended to be greater (*p* = 0.09; 12,400.0%) in CRFA pigs fed the NAB diet versus SAL pigs fed the AB diet on d 7 (Table 7). Ileal TNFα and CLAD mRNA abundance tended to be greater (*p* = 0.06 and 0.09, respectively and 34.4% and 62.0%, respectively) on d 7 in CRFA pigs fed NAB diet and SAL pigs fed AB diet when compared to SAL pigs fed NAB diet (Table 7). On d 14, jejunal GLP-2 mRNA abundances were greater (*p* = 0.03; 25.3%) in CRFA pigs fed the AB diet versus SAL pigs fed the NAB diet (Table 7). Ileal CRFR2 mRNA abundances was greater (*p* = 0.04; 19,900.0 and 14,900.0%, respectively) in CRFA pigs fed the AB diet and CRFA pigs fed the NAB diet versus SAL pigs fed the NAB diet on d 14 (Table 7). Overall, jejunal CRF mRNA abundance tended to be reduced (*p* = 0.09; 10.3%) on d 7 in CRFA pigs versus SAL pigs, regardless of dietary treatment (Table 7). On d 7, jejunal CLAD mRNA abundance was reduced (*p* = 0.02; 38.9%) in AB-fed pigs when compared to NAB-fed pigs, regardless of injection treatment (Table 7). Overall, ileal OCL mRNA abundance tended to be greater (*p* = 0.09; 50.2%) in AB-fed pigs versus NAB-fed pigs on d 7, regardless of injection treatment (Table 7). Ileal CRF mRNA abundance tended to be reduced (*p* = 0.07; 40.4%) on d 7 in AB-fed pigs when compared to NAB-fed pigs, regardless of injection treatment (Table 7). On d 14, jejunal TNFα mRNA abundance was reduced overall (*p* = 0.01; 29.7%) in AB-fed pigs versus NAB-fed pigs, regardless of injection treatment (Table 7). Ileal CRF and CRFR2 mRNA abundance tended to be greater (*p* = 0.09 and 0.06, respectively; 1,150.0% and 32.8%, respectively) on d 14 in AB-fed pigs when compared to NAB-fed pigs, regardless of injection treatment (Table 7). On d 14, ileal CRFR1 mRNA abundance was greater (*p* = 0.04; 100.0%) in AB-fed pigs when compared to NAB-fed pigs, regardless of injection treatment (Table 7). Sex differences were observed where ileal GLP-2 mRNA abundance was reduced on d 7 (*p* = 0.04) in castrated males (0.62 ± 0.15) when compared to females (1.06 ± 0.14), regardless of injection or dietary treatment (data not presented). In addition, on d 7, ileal OCL mRNA abundance tended to be reduced (*p* = 0.06) in castrated males (1.01 ± 0.26) when compared to females (1.71 ± 0.25), regardless of injection or dietary treatment (data not presented). No other intestinal mRNA abundance differences were detected (*p* > 0.10) during the dietary treatment phase with any comparison (Table 7).

**TABLE 7:** Effects of giving pigs an intraperitoneal corticotropin releasing hormone antagonist (CRFA) or sterile saline (SAL) injection during the weaning and transport process and then feeding a diet with no prophylactic antibiotics (NAB) or with prophylactic antibiotics (AB) on mRNA relative abundance of jejunal and ileal biomarkers of stress, inflammation, and intestinal on d 7 and 14 post-weaning and transport.

	SAL		CRFA			*p*-value		
Parameter	NAB	AB	NAB	AB	SEM	I^a^	D^b^	I x D
*Day 7*								
Jejunum								
TNFα	1.10	1.02	1.23	0.90	0.33	0.86	0.46	0.25
ZO-1	0.50^x^	0.71^xy^	0.64^xy^	0.80^y^	0.32	0.13	0.94	0.09
GLP-2	1.42	1.37	1.69	1.12	0.42	0.95	0.33	0.96
OCL	1.06	1.06	0.98	1.14	0.39	0.99	0.74	0.55
CLAD	0.86^y^	0.71^xy^	1.12^y^	0.50^x^	0.35	0.54	0.02	0.06
CRF	0.07	1.42	0.58	0.30	0.43	0.09	0.68	0.85
CRFR1	1.00	1.00	1.07	1.12	0.05	0.96	0.14	0.78
CRFR2	1.01^xy^	1.00^y^	1.12^x^	1.01^xy^	0.05	0.15	0.38	0.09
Ileum								
TNFα	1.44^x^	2.02^y^	1.85^y^	1.59^xy^	0.52	0.43	0.73	0.06
ZO-1	2.40	2.31	2.31	2.37	0.83	0.92	0.95	0.18
GLP-2	0.81	0.61	0.76	0.64	0.22	0.48	0.69	0.98
OCL	1.80	1.90	1.21	2.62	0.63	0.90	0.09	0.71
CLAD	1.25^x^	2.28^y^	1.77^y^	1.69^xy^	0.87	0.20	0.92	0.09
CRF	3.03	2.96	7.40	0.88	2.34	0.98	0.07	0.63
CRFR1	2.53	4.28	2.69	4.08	3.02	0.61	0.69	0.38
CRFR2	0.81	1.04	1.37	0.59	0.88	0.87	0.55	0.83
*Day 14*								
Jejunum								
TNFα	0.92	0.94	1.30	0.62	0.17	0.97	0.01	0.68
ZO-1	1.10	1.04	1.19	0.96	0.27	0.84	0.37	0.62
GLP-2	2.02^a^	2.30^ab^	2.22^ab^	2.53^b^	0.45	0.65	0.38	0.03
OCL	2.31	2.56	2.13	2.79	1.25	0.83	0.45	0.16
CLAD	1.21	0.76	1.17	0.81	0.38	0.40	0.45	0.80
CRF	1.10	1.05	1.01	1.17	0.14	0.76	0.38	0.30
CRFR1	1.05	1.00	1.00	1.10	0.07	0.58	0.25	0.98
CRFR2	1.00	1.02	1.01	1.00	0.02	0.26	0.66	0.87
Ileum								
TNFα	0.86	1.51	1.30	1.04	0.30	0.18	0.58	0.71
ZO-1	1.10	0.90	1.00	0.98	0.52	0.66	0.94	0.99
GLP-2	1.25	1.37	1.77	0.92	0.55	0.89	0.30	0.37
OCL	1.39	0.85	1.08	1.12	0.57	0.51	0.95	0.36
CLAD	0.81	1.17	1.06	0.92	0.32	0.41	0.75	0.35
CRF	1.02	1.07	1.00	1.31	0.10	0.81	0.09	0.52
CRFR1	1.10	1.00	1.15	1.59	0.11	0.51	0.04	0.43
CRFR2	1.00^c^	1.01^b^	1.14^ab^	1.20^a^	0.02	0.73	0.06	0.04

^a^ Injection treatment.

^b^ Diet treatment.

^a-c^Letters indicate significance (*p* ≤ 0.05) within a row and treatment interaction.

^x,y^Letters indicate tendencies (0.05 < *p* ≤ 0.10) within a row and treatment interaction.

#### 3.3.8 Ussing chamber parameters

**FIGURE 5:**
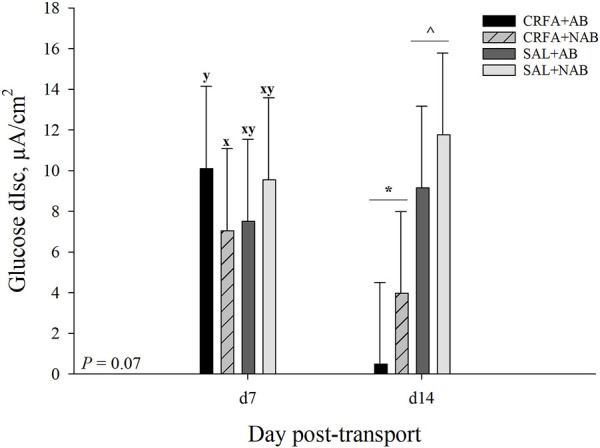
Effects of giving pigs an intraperitoneal corticotropin releasing hormone antagonist (CRFA) or sterile saline (SAL) injection on glucose change in short circuit current (dIsc) on d 7 and 14 post-weaning and transport in pigs fed no prophylactic antibiotics (NAB) or prophylactic antibiotics (AB). Data are presented as LSmeans ±1 SE. Letters^x-y^ indicate injection treatment by diet treatment tendencies within a day (0.05 < *p* ≤ 0.10). Symbols^*,^^ indicate overall injection treatment differences within a day (*p* ≤ 0.05).

On d 7, glucose dIsc tended to be reduced (*p* = 0.07; 30.3%) in CRFA pigs fed the NAB diet when compared to CRFA pigs fed the AB diet (Figure 5). On d 14, glucose dIsc was reduced overall (*p* = 0.04; 78.6%) in CRFA pigs versus SAL pigs, regardless of dietary treatment (Figure 5). No other Ussing chamber parameter differences were detected (*p* > 0.10) during the dietary treatment phase with any comparison (Table 8; Figure 5).

**TABLE 8:** Effects of giving pigs an intraperitoneal corticotropin releasing hormone antagonist (CRFA) or sterile saline (SAL) injection during the weaning and transport process and then feeding a diet with no prophylactic antibiotics (NAB) or with prophylactic antibiotics (AB) on glucose basal short-circuit current (bIsc), glucose maximum short-circuit current (mIsc), and transepithelial resistance (TER) on d 7 and 14 post-weaning and transport.

	SAL		CRFA			*p*-value		
Parameter	NAB	AB	NAB	AB	SEM	I^a^	D^b^	I x D
*Day 7*								
Basal Isc, μA/cm^2^	97.77	159.53	152.62	104.69	160.78	0.45	0.54	0.40
mIsc^c^, μA/cm^2^	84.33	140.38	134.93	89.78	98.05	0.48	0.57	0.40
TER, ohm/cm^2^	35.56	39.58	31.69	35.47	8.43	0.60	0.58	0.99
*Day 14*								
Basal Isc, μA/cm^2^	−7.74	1.36	14.95	36.03	16.92	0.08	0.35	0.72
mIsc, μA/cm^2^	3.69	10.69	19.24	36.19	14.35	0.12	0.35	0.71
TER, ohm/cm^2^	46.91	44.96	46.96	48.89	4.90	0.69	0.99	0.69

^a^ Injection treatment.

^b^ Diet treatment.

^c^ Maximum short circuit current.

## 4 Discussion

Young pigs encounter stressors such as weaning and transportation, which can result in intestinal barrier dysfunction (Spreeuwenberg et al., 2001; Boudry et al., 2004; Moeser et al., 2007a). In response to weaning and transportation stress, CRF, ACTH, and cortisol are released from the hypothalamus, anterior pituitary, and adrenal glands, respectively (Martinez et al., 1997; Taché et al., 1999; Meddings and Swain, 2000; Soderholm et al., 2002; Taché et al., 2004). As such, both cortisol and ACTH are often elevated in newly weaned and/or transported pigs (Perremans et al., 2001; Kanitz et al., 2002; Moeser et al., 2007a; Roldan-Santiago et al., 2013; Maskal et al., 2020). In agreement with these previous reports, circulating cortisol levels in the present study were greater 12 h Post-T when compared to Pre-T, regardless of the CRFA injection treatment. However, the CRFA pigs had a blunted cortisol response relative to SAL pigs. This blunted cortisol response likely indicates that the CRFA injection treatment was effective as previous research in monkeys have shown Astressin B prevents a stress-induced rise in cortisol (Berga, 2008). Although cortisol was reduced, no transport or CRFA-related ACTH differences were observed following weaning and transport in the present study. This was unexpected because ACTH is the primary regulator of cortisol secretion by the adrenal glands (Lightman et al., 2020), and previous research demonstrates that the weaning process causes an ACTH-mediated increase in basal cortisol levels (Perremans et al., 2001; Kanitz et al., 2002). Although specific reasons for the lack of weaning and transport-related ACTH differences in the present study are unclear, blood collection timing may be partly responsible. This is because blood was only collected prior to transport and 12 h after the start of transport in the present study, whereas the aforementioned reports (Perremans et al., 2001; Kanitz et al., 2002) collected blood samples immediately after the stressor (e.g., weaning) was initiated. Nevertheless, the overall decrease in circulating cortisol demonstrates that the CRFA was effective in reducing biomarkers of the physiological stress response in weaned and transported pigs.

Stress-induced immune system alterations have been previously described in pigs (Ayala et al., 2012; Hart, 2012; Escribano et al., 2015; Johnson et al., 2020). However, inconsistencies within the literature exist, with some reporting increases in immune biomarkers including IgA, IL-8, IL-1β, and LPS (Johnson, 1998; Karrow, 2006; Annamalai et al., 2015; Wyns et al., 2015), and others reporting reductions in immune biomarkers (e.g., IL-8, IL-1β) during stressful events (Tian et al., 2014). Additionally, the type and timing of the stressor has demonstrated contradictory results where cytokines (i.e., IL-1β, TNFα, and IL-6) have been increased (Johnson et al., 2002) and decreased (Goujon et al., 1995). These inconsistencies may be due to the type, duration, and intensity of the stressor or the detection method (Sorrells et al., 2009). In the present study, circulating IL-8 and IgA levels tended to be greater 12 h Post-T versus Pre-T, while circulating LPS was reduced and IL-1β tended to be reduced in pigs 12 h Post-T versus Pre-T. In the present study, the stressor was classified as “acute” because the stressor was hours versus weeks or months which is classified as “chronic” (Sorrells et al., 2009; Martínez-Miró et al., 2016) and may have augmented the response to the immune biomarkers.

During weaning and transport, feed withdrawal causes pigs to enter a state of negative energy balance (Salfen et al., 2003; Govoni et al., 2005; Inoue et al., 2005). This results in hypoglycemia that causes a drop in insulin (a potent anabolic hormone) resulting in gluconeogenesis in the liver to restore homeostatic levels of circulating glucose as well as lipolysis, which causes the release of NEFAs into circulation (Qaid and Abdelrahman, 2016). Additionally, glucocorticoids play a role in the release of stored glucose by promoting gluconeogenesis in the liver during times of low energy intake (Kuo et al., 2015). In the present study, circulating glucose and insulin were reduced and NEFAs were greater in pigs 12 h Post-T versus Pre-T. This response was likely due to the fact that weaned and transported pigs had feed withdrawn for >12 h resulting in a negative energy balance as previously described (Salfen et al., 2003; Govoni et al., 2005; Inoue et al., 2005). Additionally, circulating insulin tended to be reduced in CRFA when compared to SAL pigs. Because the CRFA treatment reduced circulating cortisol levels in the present study, and glucocorticoids cause the release of stored glucose from the liver (Kuo et al., 2015), this may explain the greater drop in circulating insulin for CRFA pigs. It is possible that CRFA pigs were more hypoglycemic throughout the weaning and transport process due to a reduced ability to mobilize energy stores resulting from inhibited cortisol release. However, these results should be interpreted with caution considering that only a numerical reduction in circulating glucose was observed for CRFA versus SAL pigs 12 h Post-T in the present study.

During periods of stress, glucagon is elevated (Yamada et al., 1993) and the production of glucose, the primary energy source for activated immune cells, is stimulated (Boden et al., 1993). As energy requirements increase, adipose tissue breaks down, and in turn, NEFAs are released into the blood (Adewuyi et al., 2005). Elevated levels of circulating NEFA are an indicator of negative energy balance as observed in dairy cattle (Bell, 1995; Herdt, 1997; Drackley, 1999). In the present study, circulating insulin tended to be reduced in CRFA pigs when compared to SAL. Overall, glucose and insulin were reduced and NEFAs were greater in pigs 12 h Post-T versus Pre-T in the present study. Literature has shown that fasting reduces insulin and glucose levels (Pollitt et al., 1982; Moebus et al., 2011) and elevates NEFA levels (Il’yasova et al., 2010). Therefore, reductions in glucose and insulin 12 h Post T was expected as pigs did not have access to feed or water during the weaning and transport process. Furthermore, previous research has demonstrated that CRF administration elevates circulating insulin levels (Genuth and Lebovitz, 1965). Therefore, by blocking CRF receptors in the present study, it was expected that insulin would be reduced in CRFA pigs and this was confirmed by the results.

Although no studies to our knowledge have reported reductive effects of Astressin B on circulating ACTH >24 h post-administration, ACTH tended to be reduced in CRFA versus SAL pigs during the dietary treatment phase in the present study, regardless of post-weaning and transport dietary treatment. However, the biological significance of this reduction is unknown and is likely minimal considering that no corresponding dietary treatment phase cortisol differences were observed between CRFA and SAL pigs. Despite the lack of dietary treatment-related circulating stress hormone differences, on d 7, jejunal CRF mRNA abundance tended to be reduced in AB-fed pigs when compared to NAB-fed pigs and tended to be decreased in CRFA versus SAL pigs. While details on the mode of action for peripheral CRF signaling remains unclear, it has been shown that stress affects the expression of CRF signaling in the gastrointestinal tract (Taché and Perdue, 2004; Larauche et al., 2009a). Reduced CRF mRNA abundance in the intestine is generally associated with a decreased stress response (Larauche et al., 2009b) and this may indicate that both AB-fed pigs and CRFA pigs were undergoing less stress when compared to NAB and SAL pigs at d 7 post-weaning and transport.

Despite the tendency for reduced jejunal CRF mRNA abundance for AB-fed pigs on d 7, the ileal mRNA abundance of CRF, CRFR1, and CRFR2 tended to be greater on d 14 in AB-fed pigs when compared to NAB-fed pigs, regardless of CRFA treatment. Additionally, CRFR2 mRNA abundance was greater in CRFA pigs fed the AB diet versus SAL pigs fed the NAB diet on d 14. Increased mRNA abundance of intestinal CRFR1 may be associated with stress-induced intestinal dysfunction (Taché et al., 2004; Larauche et al., 2008) while increased intestinal CRFR2 mRNA abundance may produce negative gastrointestinal inflammatory responses (Kokkotou et al., 2006). To our knowledge, no other studies have measured intestinal CRF, CRFR1, or CRFR2 mRNA abundance differences related to AB or CRFA administration in pigs. However, greater CRF, CRFR1, and CRFR2 mRNA and protein abundance has been observed in the jejunum of pigs 24 h after weaning stress (Moeser et al., 2007b; Smith et al., 2010; Wang et al., 2015) and this many indicate that AB-fed pigs and CRFA pigs given AB in the present study were undergoing greater stress on d 14 post-weaning and transport. However, these data should be interpreted with caution because no other corresponding physiological observations were associated with greater stress during the dietary treatment phase in AB-fed or CRFA administered pigs.

Transepithelial resistance is a measure commonly used to determine intestinal permeability with increased permeability associated with a decrease in TER (Hu et al., 2013). In the present study, no treatment related TER differences were detected at d 7 or 14 post-weaning and transport. The lack of CRFA-related TER differences conflicts with a previous report indicating that intraperitoneal CRFA administration at the same dose (30 ug/kg) results in increased TER in weaned pigs (Smith et al., 2010). However, this discrepancy may be related to the timing of TER testing in the present study as the aforementioned experiment (Smith et al., 2010) measured TER within 24 h of CRFA administration. As such, it is possible that had TER been measured within 24 h of CRFA administration in the present study, differences would have been detected. As for the lack for AB effects, no data to our knowledge are available on the direct effects of AB administration on TER in weaned pigs. However, data from the present study may suggest that AB administration does not directly impact TER, at least when measured on d 7 and 14 post-administration.

Change in short circuit current, also known as dIsc, is used to determine the active transport of substances (i.e., ions, nutrients, and drugs) through epithelial cells (Clarke, 2009), and glucose transport was evaluated following weaning and transport in the current study. In general, weaning is associated with social and environmental stress and has been shown to compromise sodium-glucose co-transporter function resulting in reduced glucose transport (Boudry et al., 2004; Li et al., 2017). It has been hypothesized that the reduction in glucose transport is mediated by the CRF signaling pathways (Li et al., 2017). As such, it would be expected that blocking CRF signaling would improve glucose active transport. Contrary to this hypothesis, glucose dIsc was reduced on d 14 by CRFA relative to SAL administration in the present study. These data may suggest that glucose active transport was inhibited by CRFA administration. However, it is important to note that this difference was only observed 14-d post-CRFA administration and thus, it is currently unclear whether this effect was directly related to an inhibition of CRFA signaling. Despite this, it appears that the inclusion of AB in the diets of weaned pigs improved glucose active transport on d 7 as indicated by a tendency for an increase in dIsc for CRFA pigs fed AB when compared to CRFA pigs fed NAB. These data may indicate that AB can play a role in improving glucose active transport in the intestine of weaned pigs, which has positive implications for nutrient uptake. However, because only a tendency was observed these data should be interpreted with caution.

The intestinal barrier consists of an epithelial cell layer joined together by tight junction proteins (Kim et al., 2012; Li et al., 2012). These proteins (i.e., ZO-1, CLAD, and OCL) regulate the leakiness of the epithelium by changing the ion and pore size (van Itallie and Anderson, 2004). Zonula occludin proteins interact directly with OCL and CLAD to regulate the assembly of the intestinal barrier (Schneeberger and Lynch, 2004; Marchiando et al., 2010). Previous studies have demonstrated that weaning stress leads to decreased jejunal OCL and CLAD protein abundance while jejunal ZO-1 protein expression is not affected (Wang et al., 2015). Other studies (Xun et al., 2018) demonstrated that jejunal OCL and ZO-1 and ileal OCL, ZO-1, and CLAD mRNA abundance is greater in less stressed late weaned versus more stressed early weaned pigs. In the present study, on d 7, CRFA pigs fed AB tended to have greater jejunal ZO-1 and reduced jejunal CLAD mRNA abundance when compared to SAL pigs fed NAB, and CRFA pigs fed NAB tended to have greater ileal CLAD when compared to SAL pigs fed AB. In addition, an overall diet treatment effect was observed whereby AB fed pigs had reduced jejunal CLAD and tended to have greater ileal OCL mRNA abundance when compared to NAB pigs on d 7. In general, these data appear to indicate that the CRFA treatment had a positive impact on tight junction protein mRNA abundance, which may explain the aforementioned reduction in Isc as an indicator of reduced intestinal permeability. To our knowledge, this is the first time that CLAD or OCL mRNA abundance differences directly related to AB administration have been observed in newly weaned and transported pigs as most studies in pigs have observed no effects (Tan et al., 2019; McConn et al., 2020; Ruckman et al., 2020; Duttlinger et al., 2021). However, research in mice suggests that AB induced disruptions in microbiota may influence intestinal barrier function (Schumann et al., 2005; Tulstrup et al., 2015; Feng et al., 2019), and this may be one mechanism by which AB altered CLAD and OCL mRNA abundance in the present study. It is important to note that while tight junction protein mRNA abundance may explain gene activations, additional research is needed to understand the assembly and structuring of the tight junction proteins.

In addition to tight junction proteins, intestinal growth and enterocyte proliferation also plays a key role in intestinal function. Glucagon-like peptide 2 is a commonly used biomarker of improved intestinal health and growth in pigs (Burrin D. et al., 2003; Burrin et al., 2005). Specifically, GLP-2 plays a role in improving nutrient absorption and gut barrier function (Burrin D. G. et al., 2003; Connor et al., 2015). In the present study, jejunal GLP-2 mRNA abundance was greater in CRFA pigs fed AB when compared to SAL pigs fed NAB on d 14. Previous research (Duttlinger et al., 2021) has demonstrated that jejunal GLP-2 mRNA abundance is increased at the end of the nursery phase in pigs provided AB immediately following weaning and transport. Therefore, it is possible that the increased GLP-2 mRNA abundance observed in the present study was directly related to the AB administration. Although, it should be noted that no GLP-2 mRNA abundance differences were observed for SAL pigs fed AB and so the CRFA treatment may have contributed to the greater GLP-2 mRNA abundance. However, the relative importance of this effect is currently unknown considering that no improvements in intestinal integrity (e.g., Isc) were observed for CRFA pigs fed AB. Additionally, since GLP-2 is produced from enteroendocrine L cells (Drucker and Yusta, 2014), an alternative explanation for the increased GLP-2 mRNA abundance observed in the present study may be directly related to either the number of enteroendocrine L cells present or the amount of GLP-2 produced per enteroendocrine L cell.

Tumor necrosis factor alpha is produced by macrophages during acute inflammation (Idriss and Naismith, 2000) and increased intestinal TNFα mRNA abundance is an indicator of greater intestinal inflammation (Pearce et al., 2014). In the present study, on d 7, ileal TNFα mRNA abundance tended to be reduced in SAL pigs fed NAB when compared to CRFA pigs fed NAB, and, on d 14, jejunal TNFα mRNA abundance was reduced in AB fed pigs versus NAB fed pigs. The effects of CRF on increasing ileal TNFα mRNA abundance in the present study contradict previous reports in *in vitro* models indicating that CRF increases TNFα leading to greater intestinal permeability (Overman et al., 2012). However, this discrepancy may be explained by response differences between *in vitro* and *in vivo* models (Nejdfors et al., 2000). As for the influence of AB on reducing jejunal TNFα mRNA abundance in the present study, these data are supported by other research (McConn et al., 2020) indicating that AB administration following weaning and transport stress decreases jejunal TNFα mRNA abundance in nursery pigs. Additional research in *in vivo* models is needed to confirm whether CRF signaling influences TNFα production in the intestine of weaned pigs.

Greater VH, reduced CD, and increased VH:CD in the small intestine are commonly reported morphological indicators of improved intestinal health and function (Piva et al., 2001; Feng et al., 2015; Xue et al., 2018). Greater VH and VH:CD ratio are associated with increased nutrient absorption (Piva et al., 2001; Feng et al., 2015) and decreased circulating proinflammatory cytokines (Willing and van Kessel, 2007; Xue et al., 2018). Additionally, reduced CD indicates a decrease in epithelial turnover in the intestine, which is associated with reduced intestinal damage and inflammation (Xue et al., 2018). Unfortunately, these morphological indicators of intestinal health are negatively impacted by weaning stress in pigs (Wang et al., 2015). In the present study, ileal CD was reduced in CRFA pigs when compared to SAL pigs on d 14 and this may have been related to the CRFA treatment blocking CRFR-1 and CRFR-2 since it is known that CRF plays a direct role in negatively impacting morphological markers of intestinal health and function (Moeser et al., 2007a). However, these results should be interpreted with caution as this was the only CRFA effect observed for intestinal morphology and this difference occurred 14 d after the CRFA injection was initially given. Additionally, on d 7, jejunal VH and CD, and ileal VH:CD ratio were greater in pigs fed AB when compared to pigs fed NAB. In general, these data are indicative of a positive benefit of AB on morphological biomarkers of intestinal health. However, it is currently unclear what role AB plays in relation to improvements in morphological indicators of intestinal health and function as variable results are reported in the literature with some studies reporting positive effects of AB on intestinal morphology including increased VH, reduced CD, and a greater VH:CD ratio (Piva et al., 2008; Oliver and Wells, 2013; Duttlinger et al., 2021), and others demonstrating no AB effects (Thymann et al., 2007; Shen et al., 2009; McConn et al., 2020). Therefore, more research should be conducted to identify the mechanism(s) by which AB influence intestinal morphology.

Stress alters immune system biomarkers and these alterations may be associated with greater or reduced levels of physiological stress that are dependent on the stressor type, duration, intensity, and detection method (Reiche et al., 2004; Sorrells et al., 2009). Because the CRFA used in the present study decreases cortisol production, it would be expected that the inhibitory effects of cortisol on pro-inflammatory cytokines would be reduced resulting in an increase in both pro-inflammatory cytokine levels and protective IgA levels as the study progressed (DeRijk et al., 1997; Rohleder et al., 2003; Elenkov, 2004; Salicrú et al., 2007). This hypothesis is supported by an observed overall increase in circulating IL-6 for CRFA pigs versus SAL pigs in the present study. A function of IL-6 is to promote cortisol release which in turn, causes an increase in the production of anti-inflammatory cytokines that act to reduce pro-inflammatory cytokines (Warren et al., 1997; Webel et al., 1997). Because CRFA inhibited cortisol production, this likely resulted in a greater increase in IL-6 due to a lack of feedback (e.g., cortisol release; Williams et al., 2009). As such, the pro-inflammatory cytokine TNFα tended to be greater in CRFA pigs fed AB when compared to SAL pigs fed AB on d 3 and 7, and also tended to be greater in CRFA pigs fed AB when compared to SAL pigs fed NAB on d 7 and 14 in the present study. Additionally, IgA was increased in CRFA pigs fed AB when compared to SAL pigs fed AB on d 7 and was greater in CRFA pigs fed NAB versus SAL pigs fed AB and CRFA pigs fed AB on d 14. This response may be related to the protective anti-inflammatory role IgA plays in the immune response (Mkaddem et al., 2014; Monteiro, 2014) and the aforementioned increase in TNFα for pigs given the CRFA injections. Finally, it was observed that circulating IL-8 was reduced in CRFA pigs fed NAB when compared to SAL pigs fed AB in the present study. This response may be explained by positive relationship between cortisol and neutrophil counts (e.g., increased cortisol = increased neutrophils; Davis et al., 1991). Therefore, because neutrophils are responsible for producing IL-8 (Lin et al., 1994), inhibiting cortisol (and potentially neutrophil counts) may have resulted in the observed decrease in circulating IL-8 for pigs given the CRFA treatment.

Low feed intake is common during the first 2 weeks post-weaning which may negatively impact growth performance (van Dijk et al., 2001). During this stressful time, the immune system is activated which uses a greater amount of energy and nutrients (Lochmiller and Deerenberg, 2000; Johnson, 2012; Kvidera et al., 2017). In the present study, ADG tended to be reduced in CRFA pigs when compared to SAL pigs from d 0–7. One explanation for the reduced growth performance may be related to the increased biomarkers of inflammatory responses observed for CRFA versus SAL pigs in the present study. It is known that circulating cytokines shift the partitioning of nutrients from growth toward the immune system (Johnson, 1998; Buchanan and Johnson, 2007; Kvidera et al., 2017). Therefore, it is possible that more energy was being diverted towards immune activation as opposed to growth in the CRFA versus SAL pigs. However, these results should be interpreted with caution as this study was not specifically designed to identify growth performance related differences related to the treatments due to individual housing of pigs and relatively low pig numbers per treatment to measure growth performance. Therefore, any future studies focused on growth performance measures related to physiological stress should take this into consideration.

The stress response related to weaning and transport has a negative impact on intestinal barrier development in pigs (Moeser et al., 2017). When the stress load imposed by the stressor is not significant (i.e., comfortable transport temperature, greater nursery pen space per pig than commercial conditions), a physiological stress response is not initiated, which may result in a lack of AB differences (McConn et al., 2020). The present study is, to the best of our knowledge, the first to report on the effects of providing a CRFA injection and AB diet to newly weaned and transported pigs. However, some limitations in the current study design should be mentioned. While the current study design was similar to previous research in newly weaned and transported pigs (Duttlinger et al., 2019; McConn et al., 2020), future studies should implement catheters to allow for more frequent blood collections to better characterize the physiological response of the pigs. In addition, the mode of the CRF receptor antagonism likely resulted in unintended downstream responses (i.e., effects on immune parameters) that should be taken into consideration when determining the timing of sample collection. Regardless, results from this study provide new information related to the interactions between the physiological stress response and AB supplementation on gastrointestinal function in pigs that may be helpful in identifying when AB supplementation is most effective or necessary in swine production.

## 5 Conclusion

Weaning and transport impact intestinal function and overall pig health and welfare. However, knowledge about how CRF signaling interacts with antibiotics to influence intestinal physiology is limited. Based on previous research, we hypothesized that CRF receptor signaling would mediate weaning and transport-induced intestinal dysfunction under production relevant conditions. In addition, we hypothesized that blocking CRF receptor signaling during the weaning process would improve measures of intestinal function similarly to the administration of commonly provided prophylactic antibiotics following weaning and transport. Results from this study indicated that, in general, CRFA pigs and pigs fed AB had some similar intestinal physiology measures post-weaning and transport. The present study brings insight into the specific mechanisms by which CRF signaling, and antibiotic supplementation may influence the intestinal physiological response in newly weaned pigs.

## Data Availability

The original contributions presented in the study are included in the article/Supplementary Material, further inquiries can be directed to the corresponding author.

## Footnotes

1 Mention of trade names or commercial products in this article is solely for the purpose of providing specific information and does not imply recommendation or endorsement by the U.S. Department of Agriculture.

2 This research was supported in part by the 2019 USDA-ARS Alternatives to Antibiotics Funding (No. 0426570), and by an appointment to the Agricultural Research Service (ARS) Research Participation Program administered by the Oak Ridge Institute for Science and Education (ORISE) through an interagency agreement between the U.S. Department of Energy (DOE) and the U.S. Department of Agriculture (USDA). ORISE is managed by ORAU under DOE contract number DE-SC0014664. All opinions expressed in this paper are the author’s and do not necessarily reflect the policies and views of USDA, ARS, DOE, or ORAU/ORISE.

3 D.P. Behan and J.E. Rivier were employed by Sentia Medical Sciences Inc. during the time this study was conducted. The remaining authors declare that the research was conducted in the absence of any commercial or financial relationships that could be construed as a potential conflict of interest.
