# Supplementary material for: Interactions between corticotropin releasing factor signaling and prophylactic antibiotics on measures of intestinal function in weaned and transported pigs
Source: Front Physiol. 2023 Oct 12;14:1266409. doi: 10.3389/fphys.2023.1266409 (PMC10615255; doi:10.3389/fphys.2023.1266409)
Supplement: Supplementary file 1 [file DataSheet1.pdf]

Supplementary Table 1. Composition of nursery diets

	Phase 1[1]		Phase 2[2]	
Item	AB	NAB	AB	NAB
Ingredient, % as fed				
Corn	30.81	31.38	37.52	38.09
SBM, 48% CP	13.95	13.95	18.00	18.00
Soybean oil	5.00	5.00	5.00	5.00
Limestone	0.79	0.79	0.74	0.74
Monocalcium phosphate	0.40	0.40	0.49	0.49
Vitamin premix[3]	0.25	0.25	0.25	0.25
Trace mineral premix[4]	0.13	0.13	0.13	0.13
Selenium premix[5]	0.05	0.05	0.05	0.05
Phytase[6]	0.10	0.10	0.10	0.10
Salt	0.25	0.25	0.25	0.25
Plasma protein	6.50	6.50	2.50	2.50
Spray dried blood meal	1.50	1.50	1.50	1.50
Soy concentrate	4.00	4.00	3.00	3.00
Select menhaden fish meal	5.00	5.00	4.00	4.00
Dried whey	25.00	25.00	25.00	25.00
Lactose	5.00	5.00	-	-
Lysine-HCl	0.07	0.07	0.20	0.20
DL-Methionine	0.22	0.22	0.23	0.23
L-Threonine	0.04	0.04	0.09	0.09
L-Tryptophan	-	-	0.01	0.01
Zinc oxide	0.38	0.38	0.38	0.38
Aureomycin 50[9]	0.40	-	0.40	-
Denagard 10[10]	0.18	-	0.18	-
Calculated chemical composition				
ME, kcal/kg	3536	3536	3510	3510
CP, %	24.62	24.62	22.87	22.87
Fat, %	7.27	7.27	7.36	7.36
SID Lys, %	1.55	1.55	1.45	1.45
Ca, %	0.90	0.90	0.85	0.85
Total P, %	0.75	0.75	0.71	0.71
Avail. P, %	0.60	0.60	0.55	0.55
Analyzed chemical composition				
CP, %	24.38	24.16	25.05	24.52
Moisture, %	9.74	9.57	9.53	9.84

Crude Fat, %	6.29	6.01	7.07	7.30
Crude Fiber, %	1.79	1.84	1.76	1.65
Ash, %	6.40	6.56	6.76	6.72
Total Lys, %	1.75	1.57	1.70	1.72
Ca, %	0.83	0.93	0.95	0.81
P, %	0.63	0.58	0.65	0.68

[1]Fed d 0 to 7 post-weaning and transport.

[2]Fed d 7 to 14 post-weaning and transport.

[3]Provided per kilogram of the diet: vitamin A, 6,614 IU; vitamin D_3, 661 IU; vitamin E, 44 IU; vitamin K, 2.2 mg; riboflavin, 9 mg; pantothenic acid, 22 mg; niacin, 33 mg.

[4]Provided available minerals per kilogram of the diet: iron, 121.3 mg; zinc, 121.3 mg; manganese, 15 mg; copper, 11.3 mg; iodine, 0.46 mg.

[5]Provided 0.3 ppm Se.

[6]Provided 600 FTU per kg of the diet.

[7]Banminth (Phibro Animal Health Corporation, Teaneck, NJ) provided 106 ppm pyrantel tartrate in the diet.

[8]Clarifly (Central Life Sciences, Schaumburg, IL) provided 9.5 ppm (Phase 1), 6.1 ppm (Phase 2), 5.4 ppm (Phase 3), and 4.7 ppm (Phase 4) diflubenzuron in the diet.

[9]Aureomycin (Zoetis, Parsippany, NJ) provided 441 ppm chlortetracycline in the diet.

[10]Denagard (Elanco Animal Health, Greenfield, IN) provided 38.6 ppm tiamulin in the diet.

Supplementary Table 2. Primers used for real time polymerase chain reactions.

Gene[1]	Sequences 5'-3' (forward/reverse)	Reference
CLAD	AGAAGATGCGGATGGCTGTC/<br>CCCAGAAGGCAGAGAGAAGC	Hu et al., 2013
GAPDH	GAAGGTCGGAGTGAACGGAT/<br>CATGGGTAGAATCATACTGGAACA	Yu et al., 2007
CRH	CCGCCAGGAGGCACCCGAGAGG/<br>GCCAAACGCACCGTTTCACTTC	Zhu et al. 2017
CRHR1	CTCATCTCCGCCTTCATCCT/<br>CCAAACCAGCACTTCTCATT	Zhu et al. 2017
CRHR2	CCGCAATGCCTACCGAGAAT/<br>TCATCCAAAATGGGCTCGCA	Li et al. 2017
TNF-α	CCCCCAGAAGGAAGAGTTTC/<br>TTGGCCCCTGAAGAGGAC	Ballweg et al., 2016
GLP-2	ACCTTGCAGCTGATGTACAC/<br>GTGTTCTCCAGGTGTGCACG	Petersen et al., 2001
OCL	ATCAACAAAGGCAACTCT/<br>GCAGCAGCCATGTACTCT	Zhang and Guo, 2009
ZO-1	AATTATCCCACAGGGAGCTATTC/<br>AGGGTTTCACCTTTCTCCTTATC	Pearce et al., 2013

Supplementary Table 3. Descriptive data of the effects of giving sentinel pigs[1] an intraperitoneal corticotropin releasing hormone antagonist (**CRFA**) or sterile saline (**SAL**) injection during the weaning and transport process on intestinal morphology and mRNA abundance post-weaning and transport.

	SAL		CRFA		
Characteristic	12 h Post-T[2]	24 h Post-T[3]	12 h Post-T	24 h Post-T	SEM
Intestinal morphology					
Jejunum					
Villus height, µm	371.69	344.39	373.93	342.15	94.99
Crypt depth, µm	170.11	157.90	162.03	165.98	20.02
VH:CD	2.18	2.16	2.32	2.02	0.53
Goblet cells, cell/mm^2	7.74	5.90	7.01	6.63	1.89
Mast cells, cell/mm^2	8.64	12.11	10.88	9.88	1.39
Ileum					
Villus height, µm	437.74	367.88	407.35	398.27	49.16
Crypt depth, µm	195.45	170.22	188.38	177.29	20.70
VH:CD	2.24	2.16	2.16	2.24	0.10
Goblet cells, cell/mm^2	16.58	16.96	14.42	19.13	2.90
Mast cells, cell/mm^2	9.21	14.38	12.08	11.50	5.28
mRNA abundance					
Jejunum					
TNF	1.23	1.25	1.31	1.15	0.21
ZO	1.02	2.51	1.55	1.74	0.76
GLP-2	1.12	2.17	2.16	0.96	0.59
OCL	1.02	0.82	1.23	0.50	0.35
CLAD	1.93	2.71	1.95	2.89	1.35
CRH	1.17	0.72	1.34	0.61	0.42
CRHR1	1.20	2.11	2.08	1.35	1.05
CRHR2	1.03	0.84	1.98	0.62	0.41
Ileum					
TNF	1.23	0.89	1.30	0.98	0.46
ZO	1.04	2.31	2.81	1.16	0.85
GLP-2	1.48	1.81	2.70	0.87	0.75
OCL	1.06	0.97	1.65	0.56	0.67
CLAD	1.81	1.05	1.67	1.26	1.19
CRH	1.94	1.78	1.68	2.10	1.49
CRHR1	1.73	2.14	3.31	0.91	1.14
CRHR2	1.79	3.52	2.03	3.10	1.51

[1]n=2/injection treatment/sex/collection time
[2]12 h Post-T, 12 h post-weaning
[3]24 h Post-T, 24 h post-weaning
